# Positioning of APOBEC3G/F Mutational Hotspots in the Human Immunodeficiency Virus Genome Favors Reduced Recognition by CD8^+^ T Cells

**DOI:** 10.1371/journal.pone.0093428

**Published:** 2014-04-10

**Authors:** Mahdis Monajemi, Claire F. Woodworth, Katrin Zipperlen, Maureen Gallant, Michael D. Grant, Mani Larijani

**Affiliations:** Immunology and Infectious Diseases Program, Division of Biomedical Sciences, Faculty of Medicine, Memorial University of Newfoundland, St. John’s, NL, Canada; Karolinska Institutet, Sweden

## Abstract

Due to constitutive expression in cells targeted by human immunodeficiency virus (HIV), and immediate mode of viral restriction upon HIV entry into the host cell, APOBEC3G (A3G) and APOBEC3F (A3F) have been considered primarily as agents of innate immunity. Recent bioinformatic and mouse model studies hint at the possibility that mutation of the HIV genome by these enzymes may also affect adaptive immunity but whether this occurs in HIV-infected individuals has not been examined. We evaluated whether APOBEC-mediated mutations within common HIV CD8^+^ T cell epitopes can potentially enhance or diminish activation of HIV-specific CD8^+^ T cells from infected individuals. We compared *ex vivo* activation of CD8^+^ T lymphocytes from HIV-infected individuals by wild type HIV peptide epitopes and synthetic variants bearing simulated A3G/F-induced mutations by measuring interferon-γ (IFN-γ) production. We found that A3G/F-induced mutations consistently diminished HIV-specific CD8^+^ T cell responses against the common epitopes we tested. If this reflects a significant trend *in vivo*, then adaptation by HIV to enrich sequences that are favored for mutation by A3G/F (A3G/F hotspots) in portions of its genome that encode immunogenic CD8^+^ T cell epitopes would favor CTL escape. Indeed, we found the most frequently mutated A3G motif (CCC) is enriched up to 6-fold within viral genomic sequences encoding immunodominant CD8^+^ T cell epitopes in Gag, Pol and Nef. Within each gene, A3G/F hotspots are more abundant in sequences encoding epitopes that are commonly recognized due to their HLA restriction. Thus, in our system, mutations of the HIV genome, mimicking A3G/F activity, appeared to abrogate or severely reduce CTL recognition. We suggest that the physiological significance of this potential effect in facilitating CTL escape is echoed in the adaptation of the HIV genome to enrich A3G/F hotspots in sequences encoding CTL epitopes that are more immunogenic at the population level.

## Introduction

APOBEC3G (A3G) and APOBEC3F (A3F) mutate deoxycytidine (dC) to deoxyuridine (dU) in the reverse transcribed nascent DNA of human immunodeficiency virus (HIV) [1]–[5]. A3G/F are constitutively expressed in CD4^+^ T lymphocytes, macrophages, and dendritic cells, the targets of HIV [6]–[12], but are also induced by interferon (IFN) in these and other cell types [11]. If a host cell expresses A3G/F, these enzymes infiltrate the virions produced by said host. In the next infection cycle, they deaminate dC in signature trinucleotide motifs (CCC and less often TCC for A3G; TTC for A3F) in the minus strand reverse-transcribed viral DNA, registering as deoxyguanosine (dG) to deoxyadenosine (dA) substitutions in the plus strand. These mutations alter reading frames, terminate translation, and/or produce mutated proteins [13]–[17]. In addition to mutating the HIV genome, A3G/F have also been reported to physically interfere with reverse transcription and viral genome integration into the host cell [18]–[20].

In the physiological setting, the effectiveness of A3G/F against HIV is restricted by several factors. First, the HIV auxiliary protein viral infectivity factor (Vif) binds to A3G/F and promotes their degradation [21]–[24]. Second, entrapment of A3G/F in high molecular mass (HMM) ribonuclear complexes renders a major fraction of the cellular pool of enzymes inactive [25]–[28]. Third, even in cells where A3G/F introduce a high mutational load into the invading viral genomes, the final mutational profile in assembled virions is limited by selection for viral fitness over successive stages of the viral life cycle, a phenomenon termed “purifying selection” [29]. As a result of these viral counter measures as well as the filtering process through selection for fitness, the majority of circulating viruses bear a lesser (sub-lethal) mutational load in their genome than would reflect the full enzymatic potential of A3G/F. Regardless of the level of their anti-viral effectiveness *in vivo*, APOBECs have classically been thought of as host restriction innate immune factors largely owing to their expression pattern and immediate modes of viral restriction. They presumably play a particularly important role in the early phases of HIV infection to limit viral propagation whilst long-lasting and more specific adaptive immune responses takes weeks to months to develop fully.

Following the initial innate immune barriers, adaptive immune responses in the form of anti-viral antibodies and cytotoxic T cells (CTL) develop and act to contain viral propagation throughout the course of infection [30]. Robust anti-viral CTL responses are associated with slower disease progression and lower viremia levels [31]–[35]. Mutations in CTL epitopes of HIV favor immune evasion by reducing CTL recognition and activation [36]–[40]. Although A3G/F are intrinsic anti-viral barriers that function early in infection, once the CTL response escalates to effective levels, the limited action of A3G/F may actually aid HIV by mutating its CTL epitopes towards immune evasion [19], [41]. In support of this possibility, the most highly mutating sites in HIV genomes mediating CTL escape are somewhat enriched in A3G hotspots [41]. However, viral genomes derived from infected individuals are inherently biased towards immune escape mutations because mutations that enhance CTL recognition would be rapidly eliminated. Mutations that might conversely improve CTL recognition, like mutations that would otherwise decrease viral fitness, would not be reflected in the isolated pool of viral genomes. On the other hand, a mouse-model study found that A3G-induced mutations outside HIV peptide epitopes can enhance the HIV-specific CTL response [42]. This study measured the response of MHC-matched CTL clones bearing transgenic peptide-specific T cell receptors (TCR) against peripheral blood mononuclear cells (PBMCs) infected by HIV produced in A3G-expressing or deficient cells. Results indicated that A3G-induced mutations outside CTL epitopes could alter the relative types of peptides available for CTL recognition. This study indicated for the first time that A3G can, in principle, modulate the CTL response by affecting the efficiency of epitope processing and presentation to CTL. However, the results of this study do not inform as to whether A3G/F mutations directly within CTL epitopes enhance or diminish the CTL response or whether the effect is measurable in the physiological setting of human HIV infection.

In this study, we simulated A3G/F mutations of CTL epitopes of HIV and measured their effect on recognition by circulating CD8^+^ T cells of HLA-matched, HIV-infected individuals *ex vivo*. We found that HIV-specific CD8^+^ T cell recognition was generally diminished by A3G/F-induced mutations. *In silico* analysis revealed the greatest enrichment of A3G/F hotspot motifs occurs within the viral genomic sequences that encode for the most immunogenic CTL epitopes. Our *ex vivo* data are consistent with the potential of A3G/F mutations to promote CTL escape. Our observation that HIV has evolved strategically-placed A3G/F mutable hotspots in its genome to maximally direct A3G/F activity towards CTL escape is further evidence that A3G/F mutations likely impact adaptive immunity *in vivo.*

## Materials and Methods

### *In silico* Demarcation and Simulation of A3G/F Hotspot Mutations in CTL Epitopes

The 9229 nucleotide-long genome of HIV isolate BRU was retrieved from the National Center for Biotechnology Information (NCBI) website. Isolate BRU was selected due to its representation of clade B, which is most prevalent in western countries [43]. Portions of the HIV genome encoding known CTL epitopes were identified using the HIV Molecular Immunology Database (http://www.hiv.lanl.gov/content/immunology/tables/ctl_summary.html). A3G/F mutations on the 5′ dG located in A3G/F hotspots in the CTL epitope-coding genomic DNA were simulated *in silico* to generate mutated epitopes. For epitopes with multiple hotspots or those with potential for the generation of novel hotspots as a result of first-round mutation, multiple mutated versions were considered. Wild type (wt) and mutant (mut) forms of all selected epitopes were synthesized to ∼90% purity (Peptide 2.0 Inc., VA-US) and used to measure peptide-specific CTL responses from HLA-matched HIV+ individuals.

### Measurement of HIV-specific CD8^+^ T Cell Recognition

HIV-infected study participants were recruited through the Newfoundland and Labrador Provincial HIV Clinic in St. John’s, Newfoundland, and selected on the basis of their HLA class I types determined for previous immunological studies [44], [45]. Informed written consent was obtained for blood collection and this study received ethical approval from the Health Research Ethics Authority of Newfoundland and Labrador for the consent and experimental protocols (reference #02.157). Cryopreserved PBMC from appropriate HLA-matched subjects were used as responder CD8^+^ T cells. The ELISPOT assay to measure the frequency of HIV-specific CD8^+^ T cells has been previously described [46]. The average of duplicate wells was obtained and the number of background spots in the negative control well subtracted. The total number of IFN-γ spot forming cells (SFC) in each well was expressed as IFN-producing SFC/10^6^ PBMC. Even though the background frequency of IFN-producing unstimulated PBMC was negligible (typically <10/10^6^ PBMC), we only considered >50 SFC/10^6^ PBMC as a positive response. (In all, 27/77 subjects tested showed a significant response.). Differences of >25% in SFC between wt and mut were considered significant.

### *In silico* Analysis of A3G/F Hotspot Frequencies in CTL Epitopes

The number of hotspots (for A3G: GGG, GGT or GGA; for A3F: GAA) inside *vs.* outside CTL epitopes in each HIV gene was determined by scanning the BRU sequence. To normalize for gene size, the frequencies of hotspot occurrence were divided by the total number of nucleotides. These frequencies were calculated both for all A3G/F hotspots, and also solely for the most frequently targeted A3G hotspot (GGG). Subsequently, the ratio (R) of normalized hotspot frequencies inside to outside epitopes was calculated for each gene. In order to evaluate differences in R amongst various genes, an index was generated by determining the average of the R of all genes and dividing the R of each gene by the average. Thus, an index of >1 indicates an above average ratio of A3G/F hotspots inside to outside CTL epitopes. We further conducted the same type of analysis within each gene in order to compare hotspot frequencies between epitopes restricted to different HLA alleles (A1, A2, A3, A11, A24, B7, B8, B35, B40, B44, B53, B57).

### Statistical Analysis

The wilcoxon signed-rank test was used to evaluate the statistical likelihood that introduction of APOBEC-related mutations into HIV CTL epitopes reduces recognition of those epitopes. Paired values for all Elispot comparisons of wt *vs.* mut epitopes were used to calculate statistical significance using Graphpad Prism 5.0.

## Results

### Demarcation of A3G/F Hotspots within Genomic Sequences Encoding CTL Epitopes and Simulation of A3G/F-induced Mutations

We identified CTL epitopes in all HIV proteins using the experimentally-verified HIV molecular immunology database (http://www.hiv.lanl.gov/content/immunology/tables/ctl_summary.html). This analysis for Gag is shown in Figure S1A (see Figure S2 for all genes). We analyzed a number of epitopes restricted to HLA-A2, B44 and B57. These HLAs were chosen for several reasons: strong CTL responses to B57-restricted epitopes correlate inversely with disease progression, and HLA-A2 and B44 are common in the population and present numerous HIV epitopes [32], [47]–[59]. Moreover, between them, these HLA alleles cover a majority of the population (http://www.allelefrequencies.net). We examined CTL epitopes in Gag, Pol and Nef because these contain the HIV peptides most prominently recognized by CD8^+^ T cells [60]–[62]. The genomic sequences of 123 epitopes from the aforementioned database were identifiable in the BRU isolate sequence (Gag shown as an example in Figure S1B; see Figure S3 for all genes). Of these, 98 epitopes (80%) contained ≥1 A3G/F hotspot in which A3G/F mutations were simulated and translated *in silico*. Epitopes for which the only possible mutation led to a stop codon or non-conservative changes in HLA anchor residues (thus impeding presentation by MHC class I) were excluded. Using these criteria, we selected and synthesized 27 wild type (wt) epitopes and 64 derivative mutants (mut) thereof (Table 1). Of the wt epitopes, 7 contained a single A3G or A3F hotspot yielding 1 mut each (example shown in Figure S1c, left panel). The other 20 contained >1 A3G/F hotspot (multiple hotspots separated by >3 nucleotides were considered independently; for example see Figure S1C, middle panel). These yielded multiple muts, some of which could occur because the initial A3G/F mutation created a new A3G/F hotspot that could be mutated again in the same or subsequent round(s) of viral replication (example shown in Figure S1C, right panel). Finally, we considered whether genomes containing these mut epitope-encoding sequences have been reported in a database of submitted HIV sequences (http://www.hiv.lanl.gov/content/sequence/QUICK_ALIGN/QuickAlign.html). We found that nearly all mut epitopes occur in the population. The selected wt and mut epitopes thus considered (Table 1) were synthesized and evaluated by ELISPOT for recognition by HIV-specific CD8^+^ T cells from HLA-matched subjects.

**Table 1:** Wild type and A3G/F-mutated epitopes used to evaluate the CD8^+^ T cell responses of HLA-matched subjects.

Epitope (sequence, origin)	Wt/Mut	HLA	DNA sequence
AISPRTLNAW Gag 146–153	Wt	B57	gccatatcacctagaactttaaatgcatgg
AISPKTLNAW	Mut	B57	gccatatcacctaaaactttaaatgcatgg
KAFSPEVIP Gag 162–170	Wt	B57	aaggctttcagcccagaagtgataccc
KAFSPKVIP	Mut	B57	aaggctttcagcccaaaagtgataccc
TSTLQEQIGW Gag 240–249	Wt	B57	actagtacccttcaggaacaaataggatgg
TSTLQKQIGW	Mut	B57	actagtacccttcaaaaacaaataggatgg
GPGVRYPLTFGWCY Nef 131–144	Wt	B57	gggccaggggtcagatatccactgacctttggatggtgctac
GPEVRYPLTFGWCY	Mut	B57	gggccagaggtcagatatccactgacctttggatggtgctac
GPEVRYPLTFRWCY	Mut	B57	gggccagaggtcagatatccactgacctttagatggtgctac
GPGVRYPLTFRWCY	Mut	B57	gggccaggggtcagatatccactgacctttagatggtgctac
GPKVRYPLTFGWCY	Mut	B57	gggccaaaagtcagatatccactgacctttggatggtgctac
GPKVRYPLTFRWCY	Mut	B57	gggccaaaagtcagatatccactgacctttagatggtgctac
GPRVRYPLTFGWCY	Mut	B57	gggccaagggtcagatatccactgacctttggatggtgctac
GPRVRYPLTFRWCY	Mut	B57	gggccaagggtcagatatccactgacctttagatggtgctac
PIVLPEKDSW Pol 398–407	Wt	B57	cctatagtgctgccagaaaaagacagctgg
PIVLPKKDSW	Mut	B57	cctatagtgctgccaaaaaaagacagctgg
FLGKIWPSHK Gag 433–442	Wt	A2	tttttagggaagatctggccttcctacaaggg
FLRKIWPSHK	Mut	A2	tttttaaggaagatctggccttcctacaaggg
FLEKIWPSHK	Mut	A2	tttttagaaaagatctggccttcctacaag
FLKKIWPSHK	Mut	A2	tttttaaaaaagatctggccttcctacaaggg
YVDRFYKTL Gag 296–304	Wt	A2	tatgtagaccggttctataaaactcta
YVDQFYKTL	Mut	A2	tatgtagaccagttctataaaactcta
PLTFGWCYKLV Nef 136–146	Wt	A2	ccactgacctttggatggtgctacaagctagta
PLTFRWCYKLV	Mut	A2	ccactgacctttagatggtgctacaagctagta
VLEWRFDSRL Nef 180–189	Wt	A2	gtgttagagtggaggtttgacagccgccta
VLEWKFDSRL	Mut	A2	gtgttagagtggaagtttgacagccgccta
FLKEKGGLEGL Nef 90–100	Wt	A2	tttttaaaagaaaaggggggactggaagggcta
FLKEKRGLEGL	Mut	A2	tttttaaaagaaaaaaggggactggaagggcta
FLKEKKGLEGL	Mut	A2	tttttaaaagaaaaaaaaggactggaagggcta
FLKKKGGLEGL	Mut	A2	tttttaaaaaaaaaggggggactggaagggcta
FLKKKRGLEGL	Mut	A2	tttttaaaaaaaaaaaggggactggaagggcta
FLKKKKGLEGL	Mut	A2	tttttaaaaaaaaaaaagggactggaagggcta
FLKKKKRLEGL	Mut	A2	tttttaaaaaaaaaaaagagactggaagggcta
FLKKKKRLKGL	Mut	A2	tttttaaaaaaaaaaaaaagactaaaagggcta
FLKKKGGLKGL	Mut	A2	tttttaaaaaaaaaggggggactaaaagggcta
FLKKKRGLKGL	Mut	A2	tttttaaaaaaaaaaaggggactaaaagggcta
FLKKKKGLKGL	Mut	A2	tttttaaaaaaaaaaaagggactaaaagggcta
FLKEKKRLEGL	Mut	A2	tttttaaaagaaaaaaaaagactggaagggcta
FLKEKKRLKGL	Mut	A2	tttttaaaagaaaaaaaaagactaaaagggcta
FLKEKGGLKGL	Mut	A2	tttttaaaagaaaaggggggactaaaagggcta
FLKEKRGLKGL	Mut	A2	tttttaaaagaaaaaaggggactaaaagggcta
FLKEKKGLKGL	Mut	A2	tttttaaaagaaaaaaaaggactaaaagggcta
ALVEICTEM Pol 188–196	Wt	A2	gcattagtagaaatttgtacagaaatgga
ALVKICTEM	Mut	A2	gcattagtaaaaatttgtacagaaatgga
LLWKGEGAV Pol 956–964	Wt	A2	ctcctctggaaaggtgaaggggcagta
LLWKSEGAV	Mut	A2	ctcctctggaaaagtaaaggggcagta
LLWKSERAV	Mut	A2	ctcctctggaaaagtgaaagggcagta
LLWKSEEAV	Mut	A2	ctcctctggaaaagtgaagaggcagta
ILKEPVHGV Pol 464–472	Wt	A2	attctaaaagaaccagtacatggagtgtat
ILKEPVHRV	Mut	A2	attctaaaagaaccagtacatagagtgtat
VIYQYMDDL Pol 334–342	Wt	A2	gttatctatcaatacatggatgatttg
VIYQYIDDL	Mut	A2	gttatctatcaatacatagatgatttg
VLVGPTPVNI Pol 131–140	Wt	A2	gtattagtaggacctacacctgtcaacata
VLVRPTPVNI	Mut	A2	gtattagtaagacctacacctgtcaacata
IYQYMDDLYV Pol 335–344	Wt	A2	atctatcaatacatggatgatttgtatgta
IYQYIDDLYV	Mut	A2	atctatcaatacatagatgatttgtatgta
LLRWGLTTPDKK Pol 364–375	Wt	A2	ctgttgaggtggggacttaccacaccagacaaaaaa
LLKWGLTTPDKK	Mut	A2	ctgttgaagtggggacttaccacaccagacaaaaaa
LVGPTPVNII Pol 132–141	Wt	A2	ttagtaggacctacacctgtcaacataatt
LVRPTPVNII	Mut	A2	ttagtaagacctacacctgtcaacataatt
VLDVGDAYFSV Pol 263–273	Wt	A2	gtactggatgtgggtgatgcatatttttcagtt
VLDVSDAYFSV	Mut	A2	gtactggatgtaagtgatgcatatttttcagtt
LSEGATPQDL Gag 175–184	Wt	B44	ttatcagaaggagccaccccacaagattta
LSERATPQDL	Mut	B44	ttatcagaaagagccaccccacaagattta
LSKRATPQDL	Mut	B44	ttatcaaaaagagccaccccacaagattta
LSKGATPQDL	Mut	B44	ttatcaaaaggagccaccccacaagattta
RDYVDRFYKTL Gag 294–304	Wt	B44	agagactatgtagaccggttctataaaactcta
RDYVDQFYKTL	Mut	B44	agagactatgtagaccagttctataaaactcta
KEKGGLEGL Nef 92–100	Wt	B44	aaagaaaaggggggactggaagggcta
KEKGGLERL	Mut	B44	aaagaaaaggggggactggaaaggcta
KEKGGLKRL	Mut	B44	aaagaaaaggggggactaaaaaggcta
KEKGGLKGL	Mut	B44	aaagaaaaggggggactgaaagggcta
KEKKRLEGL	Mut	B44	aaagaaaaaaggggactggaagggcta
KEKRRLEGL	Mut	B44	aaagaaaagagaagactggaagggcta
PPIPVGEIY Gag 254–262	Wt	B35	ccacctatcccagtaggagaaatttat
PPIPVREIY	Mut	B35	ccacctatcccagtaagagaaatttat
PPIPVRKIY	Mut	B35	ccacctatcccagtaagaaaaatttat
PPIPVKKIY	Mut	B35	ccacctatcccagtaaaaaaaatttat
PPIPVGKIY	Mut	B35	ccacctatcccagtaggaaaaatttat
PPIPVEKIY	Mut	B35	ccacctatcccagtagaaaaaatttat
TVLDVGDAY Pol 262–270	Wt	B35	acagtactggatgtgggtgatgcatat
TVLDVSDAY	Mut	B35	acagtactggatgtaagtgatgcatat
GPGVRYPLTF Nef 130–139	Wt	B35	gggccaggggtcagatatccactgaccttt
GPRVRYPLTF	Mut	B35	gggccaagggtcagatatccactgaccttt
GPEVRYPLTF	Mut	B35	gggccagaggtcagatatccactgaccttt
GPKVRYPLTF	Mut	B35	gggccaaaggtcagatatccactgaccttt
YPLTFGWCY Nef 134–143	Wt	B35	tatccactgacctttggatggtgctac
YPLTFRWCY	Mut	B35	tatccactgacctttagatggtgctac
VPLDEDFRKY Pol 273–382	Wt	B35	gttcccttagatgaagacttcaggaagtat
VPLDKDFRKY	Mut	B35	gttcccttagataaagacttcaggaagtat
VPLDEDFKKY	Mut	B35	gttcccttagatgaagacttcaaaaagtat
VPLDKDFKKY	Mut	B35	gttcccttagataaagacttcaaaaagtat

Wild type epitopes and A3G/F-mutated versions thereof used to test the HIV-specific CD8^+^ T cell response of HLA-matched subjects by ELISPOT are shown. The polypeptide of origin for each epitope, its restricting HLA allele and the amino acid position of the epitope in HIV-1 Bru are indicated.

### CD8^+^ T Cell Recognition of HLA-B57-restricted Epitopes is Diminished by A3G/F-simulated Mutations

**Figure 1:**
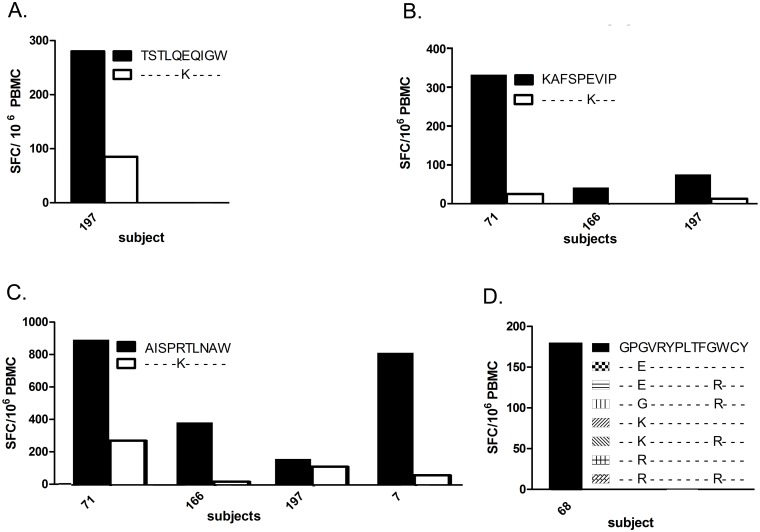
CD8^+^ T cell response to HLA-B57-restricted wild type and A3G/F-mutated epitopes. Responses of PBMC from HLA-matched subjects to wild type and mutant epitopes are shown on the Y-axis, as IFN-γ SFC (Spot Forming Cells)/10^6^ PBMC. **A.** Comparison of IFN-γ induction between wild type and a mutant form of the B57-restriced Gag epitope TSTLQEQIGW. **B.** Comparison of IFN-γ induction between wild type and 2 mutant forms of the B57-restriced Gag epitope KAFSPEVIP. **C.** Comparison of IFN-γ induction between wild type and a mutant form of the B57-restriced Gag epitope AISPRTLNAW. **D.** Comparison of IFN-γ induction between wild type and 7 mutant forms of the B57 restricted Nef epitope GPGVRYPLTFGWCY.

We compared the CD8^+^ T cell response against 3 HLA-B57-restricted epitopes in Gag and the corresponding variants with APOBEC-induced mutations. Of 5 HLA-B57^+^ subjects (P7, P45, P71, P166, P197) tested against TSTLQEQIGW and 1 mut, P197 alone had a positive response that was 3-fold higher against the wt peptide (280 *vs.* 85 SFC/10^6^ PBMC) (Figure 1a). Three of the same 5 subjects had positive responses against KAFSPEVIP (P71: 397; P166: 50; P197: 90 SFC/10^6^ PBMC), whilst none had responses against the mut (Figure 1b). Eight subjects (P7, P20, P45, P71, P76, P166, P185, P197) were tested for responses against AISPRTLNAW and 1 mut. Four (P7, P71, P166, P197) had positive responses. P7 and P166 responded only against the wt peptide, while P71 and P197 exhibited responses that were 3- and 1.4-fold higher against wt (P71: 895 *vs.* 273; P197: 160 *vs.* 112 SFC/10^6^ PBMC) (Figure 1c). Responses against the Nef epitope GPGVRYPLTFGWCY and 7 muts were compared in 6 subjects (P20, P45, P68, P76, P166, P185). Only P68 had a response, and only against the wt peptide (Figure 1d). Taken together, 9 HLA-B57 subjects responded only against the wt epitopes, whilst 3 responded against both the wt and corresponding mut for which the responses against wt epitopes were 1.4- to 3-fold higher. Thus, for the HLA-B57-restricted epitopes and subjects we studied, introduction of signature A3G/F-induced mutations into the epitopes consistently abrogated or reduced CD8^+^ T cell recognition.

### A3G/F-simulated Mutations Diminish CD8^+^ T Cell Recognition of HLA-A2-restricted Epitopes

**Figure 2:**
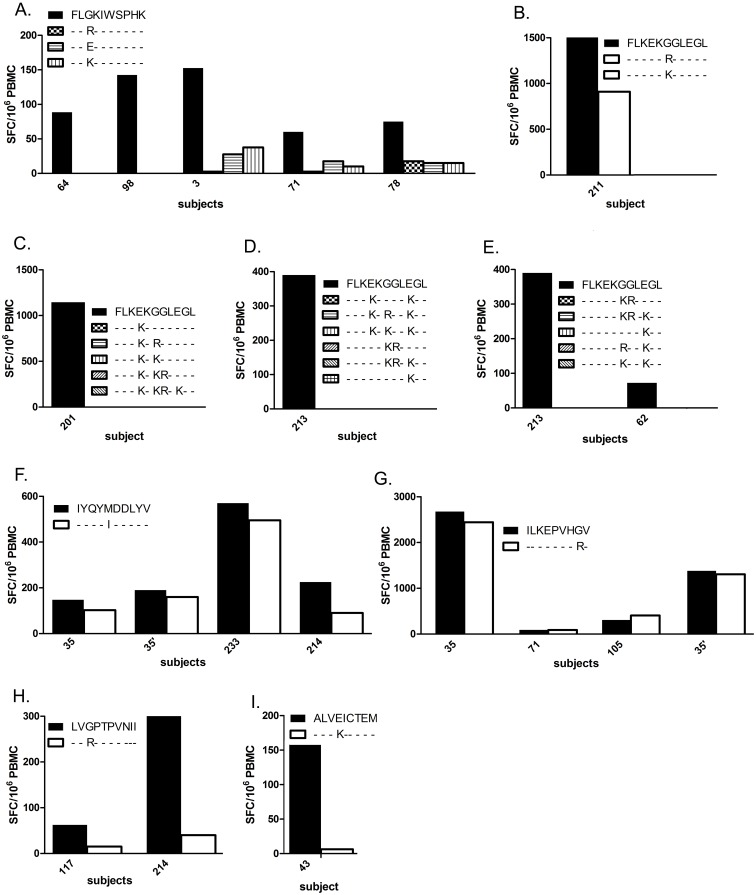
CD8^+^ T Cell response to HLA-A2-restricted wild type and A3G/F-mutated epitopes. Responses of PBMC from HLA-matched subjects to wild type and mutant epitopes are shown on the Y-axis, as IFN-γ SFC/10^6^ PBMC. **A.** Comparison of IFN-γ induction between wild type and 3 mutant forms of the HLA-A2 restricted Gag epitope FLGKIWSPHK. **B-E.** Comparison of IFN-γ induction between wild type and 15 mutant forms of the HLA-A2 restricted Nef epitope FLKEKGGLEGL. **F.** Comparison of IFN-γ induction between wild type and a mutant form of the HLA-A2 restricted Pol epitope IYQYMDDLYV. **G.** Comparison of IFN-γ induction between wild type and a mutant form of the HLA-A2 restricted Pol epitope ILKEPVHGV. **H.** Comparison of IFN-γ induction between wild type and a mutant form of the HLA-A2 restricted Pol epitope LVGPTPVNII. **I.** Comparison of IFN-γ induction between wild type and a mutant form of the HLA-A2 restricted Pol epitope ALVEICTEM.

We measured recognition of the HLA-A2-restricted Gag epitope FLGKIWPS and 3 muts by 9 subjects (P3, P18, P30, P35, P64, P71, P78, P98, P234). Five had positive responses exclusively against the wt peptide (Figure 2a). Recognition of the immunodominant Nef epitope FLKEKGGLEGL epitope and 15 muts was assessed in 30 subjects, of whom 4 responded against the wt peptide. Of these, P211 responded against a single mut with 1.6-fold less magnitude than against the wt peptide (1512 *vs.* 910 SFC/10^6^ PBMC) (Figure 2b-e). Recognition of 4 HLA-A2-restricted Pol epitopes and corresponding variants was compared. The Pol epitope IYQYMDDLYV and 1 mut were tested in 20 subjects, of whom 3 responded with moderately higher responses against the wt than mut peptides (for wt *vs.* mut: P35: 147 *vs.* 92; P35′: 190 *vs.* 160; P214: 225 *vs.* 90; P233: 570 *vs.* 495 SFC/10^6^ PBMC) (Figure 2f). Recognition of ILKEPVHGV and 1 mut was tested in 17 subjects, of whom 3 responded, comparably against wt and mut peptides (for wt *vs.* mut: P35: 2675 *vs.* 2442; P35′: 1380 *vs.* 1305; P71: 90 *vs.* 88; P105: 305 *vs.* 405 SFC/10^6^ PBMC) (Figure 2g). LVGPTPVNII and 1 mut were tested in 8 subjects, with 2 responding only against wt (Figure 2h). ALVEICTEM and 1 mut were tested in 11 subjects and only the wt peptide was recognized in 1 subject (Figure 2i). Altogether, for 6 HLA-A2-restricted epitopes in Gag, Nef and Pol, 4 were better recognized by CD8^+^ T cells as wt peptides, whilst 2 were recognized comparably well as wt or mut peptides. A3G/F-simulated mutations in HLA-A2-restricted epitopes thus diminished recognition by CD8^+^ T cells in the majority of cases.

### A3G/F-simulated Mutations Reduce the CD8^+^ T cell Response to the Majority of HLA-B44-restricted Epitopes

**Figure 3:**
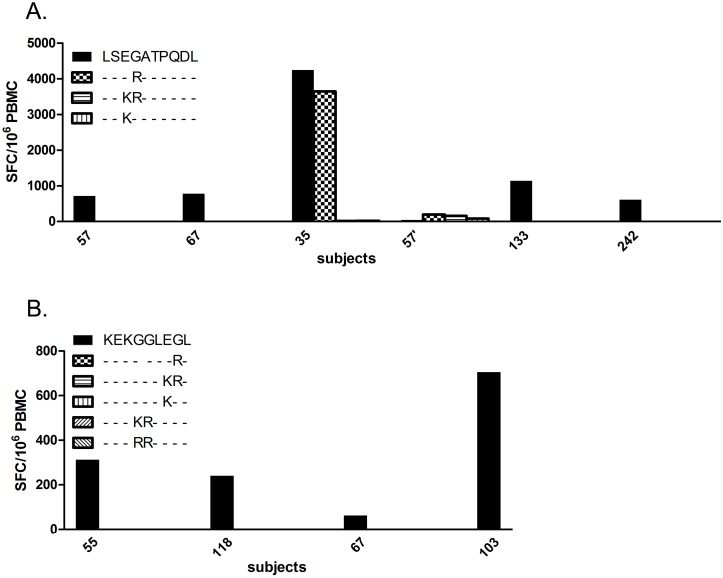
CD8^+^ T cell response to HLA-B44-restricted wild type and A3G/F-mutated epitopes. Responses of PBMC from HLA-matched subjects to wild type and mutant epitopes are shown on the Y-axis as IFN-γ SFC/10^6^ PBMC. **A.** Comparison of IFN-γ induction between wild type and 3 mutant forms of the HLA-B44 restricted Gag epitope LSEGATPQDL. **B.** Comparison of IFN-γ induction between wild type and 6 mutant forms of the HLA-B44 restricted Nef epitope KEKGGLEGL.

Recognition of 2 HLA-B44-restricted wt epitopes and corresponding muts in Gag was examined. Sixteen HLA-B44**^+^** subjects were tested for CD8^+^ T cell responses against LSEGATPQDL and 3 muts. Five responded against the wt peptide (P35: 4245; P57: 712; P67: 775; P133: 1137; P242: 610 SFC/10^6^ PBMC) and 2 had lesser responses against the G to R mut (P35: 3645; P57: 192 SFC/10^6^ PBMC) (Figure 3a). Five subjects were tested for CD8^+^ T cell recognition of the Nef epitope KEKGGLEGL and 5 muts, 4 of whom responded to wt only (Figure 3b). Overall, 9 subjects responded to 2 epitopes restricted to HLA-B44. Of these, 7 responded only to wt, whilst 2 responded to both wt and mut. In all cases, the response to wt was significantly higher. Therefore, for the HLA-B44-restricted epitopes and subjects we studied, introduction of signature A3G/F mutations into the epitopes consistently abrogated or reduced CD8^+^ T cell recognition. In order to evaluate the statistical significance of our results, we conducted a Wilcoxon signed-rank test, considering the Elispot results obtained for all HLA-57, A2- and B44- restricted wt/mut pairs. We obtained a p-value of 0.0001, which supports statistical significance of our finding that the response to mut epitopes was diminished as a result of APOBEC-simulated mutations.

### Analysis of A3G/F Hotspot Frequency inside CTL Epitopes

**Figure 4:**
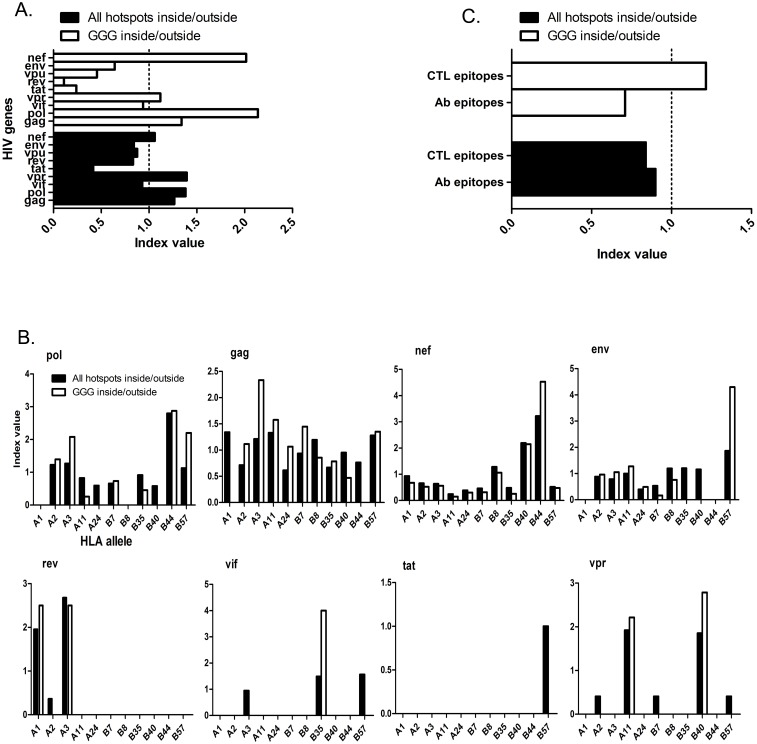
Relative frequency of A3G/F hotspot motifs inside *vs.* outside CTL epitopes in HIV genes. **A.** For each HIV gene, the frequency of A3G/F hotspots (either all hotspots: GGG, GGA, GGT, GAA, or GGG alone) was calculated in genomic sequences that encode CTL epitopes and sequences that do not encode for CTL epitopes. Normalized frequencies were calculated by taking into account the total nucleotide length of the sequence. For each HIV gene, the normalized frequency of A3G/F hotspots inside sequences that encode CTL motifs was divided by the normalized frequency of A3G/F hotspots in sequences that do not encode CTL epitopes to generate a Ratio (R-value). The average of R-values amongst all HIV genes was determined and each R-value was divided by the average to obtain the index value, which reflects enrichment of hotspots within CTL epitopes, relative to other HIV genes. **B.** The same analysis as panel A was conducted within each HIV gene to compare the relative enrichment of A3G/F hotspots in sequences that encode CTL epitopes restricted to different HLA alleles. **C.** Comparison of A3G/F enrichment in CTL epitopes *vs.* the portions of the Env gene that encode antibody recognition epitopes. Average R-value for the CTL epitopes of all genes (average of all R values from panel A) was compared to the R value of A3G/F hotspots inside/outside sequences encoding antibody recognition motifs within Env. Index value of 1 is shown as a scale because an index >1 indicates relative enrichment.

The *ex vivo* reduction of HIV-specific CD8^+^ T cell responses by A3G/F mutations introduced into common HLA-A2, B44 and B57-restricted epitopes suggests that it would be advantageous for HIV to adapt towards maximizing A3G/F hotspots in genomic sequences encoding highly immunogenic or broadly presented CTL epitopes. To test this, we calculated the ratio (R) of A3G/F hotspot frequency inside *vs.* outside CTL epitopes within each HIV gene, normalizing for sequence length. We considered the frequencies of all A3G/F hotspots, but because A3G mutates HIV several fold more potently than A3F, and CCC (GGG on the plus DNA strand) is its preferentially targeted motif [13], [27], [63], [64], we also considered the frequency of GGG independently (table S1). Relative enrichment of hotspots inside *vs.* outside CTL epitopes for each gene is reflected by an index that constitutes the ratio of the R value of each gene to the average R of all genes. Considering the index values for all hotspots, as well as GGG alone, Gag, Pol and Nef exhibited the highest indices (for GGG: 1.3, 2.1 and 2.0 respectively) (Figure 4a).

We next addressed the suggestion that enrichment of A3G/F hotspots amongst various epitopes in each gene correlates with immunogenicity, as defined by the ability to elicit a robust (or broad, at the population level) CTL response. To examine this, we categorized epitopes on the basis of their restricting HLA (A1, A2, A3, A11, A24, B7, B8, B35, B44, B57). For each gene, we tallied hotspots inside *vs.* outside epitopes restricted to each HLA allele (Figure 4b). We observed a strong trend. For genes with the highest number of experimentally verified CTL epitopes (Gag, Pol, Env and Nef), the indices for HLA-B57 were >1, with GGG being over-represented by 1.7- to 4.9-fold. In contrast, the frequency of GGG motifs in HLA-B35 was >2-fold lower than that of other HLA-B alleles. The most striking enrichment was observed in Nef, where all A3G/F hotspots and GGG were respectively 5.7- and 17.6-fold enriched in B44-restricted as compared to B35-restricted epitopes. Nef contains a single B44-restricted epitope that induces robust CTL responses in a broad proportion of the population [32]. We noted that its coding sequence can potentially generate >20 muts. We also noted a higher frequency of GGG motifs in A3-restricted epitopes of Gag and Pol (2.1- to 2.3-fold enriched over epitopes restricted to other HLAs). These results may reflect the evolution of the viral genome in line with a higher number of CTL epitopes in these gene segments as well as the prevalence of the A3 allele in the population.

In order to test if there is broad A3G/F hotspot enrichment within CTL epitopes, we compared the R of A3G/F hotspots inside to outside CTL epitopes of each gene to the R of hotspots inside to outside antibody epitopes in Env, which we considered as a control. The R for antibody epitopes in Env is 0.71 and 0.9 for all A3G/F hotspots or GGG, respectively. These are 1.3- to 3-fold lower than the R values for CTL epitopes of Pol (1.06 and 1.63), Gag (1.16 and 2.60) and Nef (0.89 and 2.45), but interestingly, up to 7-fold higher than the R values found in the CTL motifs of other HIV genes (Vif, Vpr, Tat, Rev and Vpu). The average R value of GGG inside to outside CTL epitopes in all genes is 2-fold higher than its counterpart for Ab epitopes across all genes (Figure 4c). Collectively, this analysis suggests 2 levels of enrichment of A3G/F hotspots of significance for viral escape from the CTL response: first, enrichment in CTL epitopes of Gag, Pol and Nef, and second, enrichment in epitopes within each gene restricted to HLAs that present more immunogenic epitopes (e.g. HLA-B57) and HLAs that occur more broadly at the population level (e.g. HLA-B44, A3).

## Discussion

The anti-HIV response by CD8^+^ CTL plays a key role in controlling viremia as illustrated by the correlation between disease progression and class I HLA genotype [47], [49], [65]–[68]. A mouse model study found that A3G-induced mutations can potentially improve the CTL response against HIV-infected cells *in vitro* by producing truncated peptides that are more effectively processed and presented [42]. In contrast, another study proposed that A3G hotspots in CTL escape sites reduce CTL recognition of HIV [41]. It is now appreciated that A3G/F may benefit HIV by contributing to drug resistance and other contextual fitness-enhancing mutations [19], [69], [70]. Here we show that A3G/F mutations consistently compromise adaptive immune responses in HIV-infected individuals by favoring CTL escape. Nevertheless, since we did not directly test the effect of random mutations in this study, we cannot conclude that A3G/F mutations specifically diminish the CTL response, nor can we extend our observations beyond the known set of CTL epitopes that have been identified in HIV, to date. However, we note that multiple previous studies of the CD8^+^ T cell response in HIV+ cohorts to epitope variants adapted at the population level found that >50% of mut epitopes including hypothetical variants (*i.e.* like our muts: not sequenced from the HIV+ individual whose CTL response was tested) retained their ability to stimulate CD8^+^ T cells [35], [44], [45].

Several non-mutually exclusive scenarios can account for our observations that in our system the simulated A3G/F-induced reduced CD8^+^ T cell recognition of HIV. First, it is possible that some *in silico*-simulated A3G/F mutations may not have arisen in the subjects we tested. This is unlikely since we verified that all of our mut epitopes have been reported at the population level in a database of HIV CTL epitope variants (http://www.hiv.lanl.gov/content/sequence/QUICK_ALIGN/QuickAlign.html). Even if the occurrence of the mutation elicited a response in as low as ∼1 in 10,000 CD8^+^ T cells, it should be detectable by ELISPOT. It is possible that the decrease in CTL response to mut epitopes was a product of our particular experimental procedures. Our finding that several mut epitopes within Pol were equally well recognized (Figure 2f, g) argues against this. Finally, it is possible that our observations reflect the physiological effect of A3G/F-induced mutations within CTL epitopes. In support of this, our observation that A3G/F hotspots are selectively enriched in CTL epitope-encoding portions of the HIV genome indicates that the reduced CTL recognition of A3G/F-mutated epitopes represents an *in vivo* phenomenon pronounced enough to leave an evolutionary footprint on the viral genome.

The most direct interpretation of our data is that the majority of A3G/F mutations within CD8^+^ T cell epitopes reduce peptide recognition by CD8^+^ T cells originally activated by the wild type HIV peptide epitope. Cross-reactive recognition of the mutated peptide seems unlikely in the case of non-conservative amino acid substitutions associated with A3G/F mutations such as G to R. If the mutated peptide were itself immunogenic and present at high enough levels, then a non-cross-reactive set of CD8^+^ T cells would be expected to arise against the new epitope. The reduced but apparent reactivity with some mutated epitopes could thus stem either from partial cross-reactivity of CD8^+^ T cells activated by the wild type epitope or from a smaller, non-overlapping CD8^+^ T cell response selectively activated by the new, A3G/F-mutated epitope. Studies of the T cell response at the clonal level and of endogenous viral sequences found in each HIV+ individual would be required to further address the issue of cross-reactivity *vs.* activation of a non-overlapping set of T cells against a *de novo* A3G/F-derived epitope. Based on previous studies, cross-reactivity is quite likely in the case of conservative or semi-conservative mutations introduced into epitopes by A3G/F [35]. Rare conservative A3G/F-induced mutations, such as the M to I mutation in the HLA A2-restricted epitope IYQYMDDLYV, were more likely to elicit comparable responses. It is probable that A3G/F-mutated epitopes are less efficient at activating CTL “selected” to recognize the wt epitope due to non-conservative mutations and the effect thereof on recognition by the T cell receptor. In the setting within which we tested the impact of A3G/F mutations on activation of CD8^+^ T cells from HIV-infected individuals, reduction or abrogation of CD8^+^ T cell activation was the rule.

The significance of our results lies in demonstration of viral access to multiple avenues of mutational escape from immune recognition. Infidelity and lack of proof-reading for reverse transcription results in 1 to 2 mutations/viral genome/round of replication [71]–[75]. While this level of mutation ensures that escape mutations occur at the level of the overall virus population, it would still leave the majority of viruses susceptible until selective expansion of the mutated virus allowed it to become predominant. The need to achieve expeditious escape from a diverse T cell response raises a higher mutational barrier. In this context, A3G is a highly processive enzyme that by conservative estimates, can introduce tens of mutations in a viral genome in a single replication round, and hundreds of mutations in a viral genome in a replication round when its activity is unhindered by Vif [13], [76]. Indeed, turning off (and likely also turning down) the expression and/or A3G-restricting effectiveness of Vif has been shown to be a means by which HIV fine-tunes the increase in A3G activity in order to overcome other selective pressures such as anti-viral drugs [77], [78]. Taken together with selective positioning of A3G motifs within CD8^+^ T cell epitopes over most other portions of the HIV genome, this provides HIV with a substantially more robust mechanism of generating mutations that affect immune recognition. Furthermore, the specificity of A3G/F, which often led to non-conservative amino acid changes in CTL epitopes that we examined, positively skews the likelihood of mutation contributing to immune escape.

Our findings contrast with those of a recent mouse model study showing that A3G mutations promote the CTL response through introducing stop codons leading to the generation of truncated proteins [42]. This study demonstrated the potential of A3G/F to increase HIV immunogenicity through an indirect mechanism involving mutation of sequences outside of actual CTL epitopes, which modulates the efficacy of epitope production and/or presentation. Here we report that the direct action of A3G/F within sequences encoding CD8^+^ T cell epitopes most often has the opposite effect in HIV-infected individuals. Whilst the mouse study utilized transgenic T cell receptor-bearing T cells in a well defined animal model and elegantly showed that APOBEC action can impact the CTL response, the work presented here takes into account a diverse set of human subjects with respect to differences in HLA makeup, anti-viral drug treatment and stage of HIV infection. In this context, we note that our data reflect a general physiological effect of A3G/F-induced mutations within CTL epitopes for the cohort of HIV-infected subjects we studied. What exceptions there may be at the individual level remains to be tested. Our observation that A3G/F hotspots are enriched in CTL epitope-encoding sequences of the HIV genome argues that the magnitude of CTL escape as a result of A3G/F-induced mutations is significant enough to have left its evolutionary footprint on the viral genome. Further work is required to shed light on the molecular and cellular pathways through which these DNA-mutating enzymes alter CTL recognition of infected cells, as well as to better understand the role of A3G/F-induced mutations outside CTL epitopes in affecting the processing and presentation of HIV peptides to T cells.

## Supporting Information

Figure S1 Delineation of the amino acid and DNA sequences of CTL epitopes. **A.** locations of CTL epitopes in Gag, in the HIV-1 Bru isolate sequence of HIV. The brackets indicate epitopes on the peptide sequence. Epitopes are shown in different colors depending on HLA allele restriction, which is indicated above the bracket. Dashed lines display epitopes that are devoid of A3G/F hotspots and continuous lines show epitopes that harbor A3G/F hotspots. **B.** locations of the viral genomic sequences that encode CTL epitopes in Gag in the HIV-1 Bru isolate plus-sense strand. Colors delineate the sequences encoding CTL epitopes from the surrounding DNA, but do not otherwise correspond to any common features amongst genes or epitopes. **C.** Simulation of A3G/F-mediated mutations of CTL epitopes. Wild-type epitopes and their encoding DNA sequence are shown. A3G/F targeting hotspots in the plus-sense epitope encoding sequence are underlined and colored in blue. Simulated A3G/F-mediated G to A mutations in the viral genomic sequence and the resulting amino acid mutations in CTL epitopes are shown below in red. Left panel shows a typical epitope with a single possible mutation. Middle panel shows a typical epitope with Multiple independent mutations. Hotspots separated by >3 nucleotides were considered as independent and mutant epitopes bearing either mutation or combinations of multiple mutations were considered. Right panel shows a typical epitope with multiple sequential mutations. For hotspots where an initial A3G/F-mediated mutation can generate a new A3G/F hotspot which may be mutated in the same or a subsequent replication cycle, variants bearing different combinations of mutations were considered.
(PDF)

Figure S2 Map of all HIV-1 Bru isolate CTL epitopes across the viral proteome. HIV proteins are shown individually (**A:** Pol, **B:** Rev, **C:** Vif, **D:** Nef, **E:** Vpr, **F:** Env, **G:** Gag, **H:** Tat). The brackets indicate epitopes on the peptide sequence. Epitopes restricted to different HLA alleles are shown in different colors and the restricting HLA is indicated above the bracket. Broken lines display epitopes with no A3G/F hotspots and continuous lines show epitopes that harbor A3G/F hotspots.
(PDF)

Figure S3 Map of the viral genomic sequences that encode CTL epitopes in the HIV-1 Bru isolate plus-sense coding DNA. Sequence of each gene is shown (**A:** Pol, **B:** Rev, **C:** Vif, **D:** Nef, **E:** Vpr, **F:** Env, **G:** Gag, **H:** Tat). Colors differentiate sequences encoding CTL epitopes from the surrounding DNA. Colors do not otherwise correspond to any common features amongst genes or epitopes.
(PDF)

Table S1 Relative enrichment of A3G/F hotspot inside/outside CTL epitopes in HIV genes. For each HIV gene, the frequency of A3G/F hotspots (either all hotspots: GGG, GGA, GGT, GAA, or GGG alone) was calculated in genomic sequences that encode for CTL epitopes and those that do not encode for CTL epitopes. Normalized frequencies were calculated by taking into account the total nucleotide length of the sequence. For each HIV gene, the normalized frequency of A3G/F hotspots inside sequences that encode CTL motifs was divided by the normalized frequency of A3G/F hotspots in sequences that do not encode CTL epitopes to generate a Ratio (R-value). The average of R-values was determined and each R-value was divided by the average to obtain the index value and allow for determination of the relative enrichment of hotspots within the CTL epitopes of each gene, as compared to the average ratio of hotspots inside to outside CTL epitopes. An index value >1 indicates relative enrichment.
(PDF)
