# Supplementary material for: Positioning of APOBEC3G/F Mutational Hotspots in the Human Immunodeficiency Virus Genome Favors Reduced Recognition by CD8+ T Cells
Source: PLoS One. 2014 Apr 10;9(4):e93428. doi: 10.1371/journal.pone.0093428 (PMC3982959; doi:10.1371/journal.pone.0093428)
Supplement: Figure S1 — Delineation of the amino acid and DNA sequences of CTL epitopes. A. locations of CTL epitopes in Gag, in the HIV-1 Bru isolate sequence of HIV. The brackets indicate epitopes on the peptide sequence. Epitopes are shown in different colors depending on HLA allele restriction, which is indicated above the bracket. Dashed lines display epitopes that are devoid of A3G/F hotspots and continuous lines show epitopes that harbor A3G/F hotspots. B. locations of the viral genomic sequences that encode CTL epitopes in Gag in the HIV-1 Bru isolate plus-sense strand. Colors delineate the sequences encoding CTL epitopes from the surrounding DNA, but do not otherwise correspond to any common features amongst genes or epitopes. C. Simulation of A3G/F-mediated mutations of CTL epitopes. Wild-type epitopes and their encoding DNA sequence are shown. A3G/F targeting hotspots in the plus-sense epitope encoding sequence are underlined and colored in blue. Simulated A3G/F-mediated G to A mutations in the viral genomic sequence and the resulting amino acid mutations in CTL epitopes are shown below in red. Left panel shows a typical epitope with a single possible mutation. Middle panel shows a typical epitope with Multiple independent mutations. Hotspots separated by >3 nucleotides were considered as independent and mutant epitopes bearing either mutation or combinations of multiple mutations were considered. Right panel shows a typical epitope with multiple sequential mutations. For hotspots where an initial A3G/F-mediated mutation can generate a new A3G/F hotspot which may be mutated in the same or a subsequent replication cycle, variants bearing different combinations of mutations were considered. (PDF) [file pone.0093428.s001.pdf]

A.

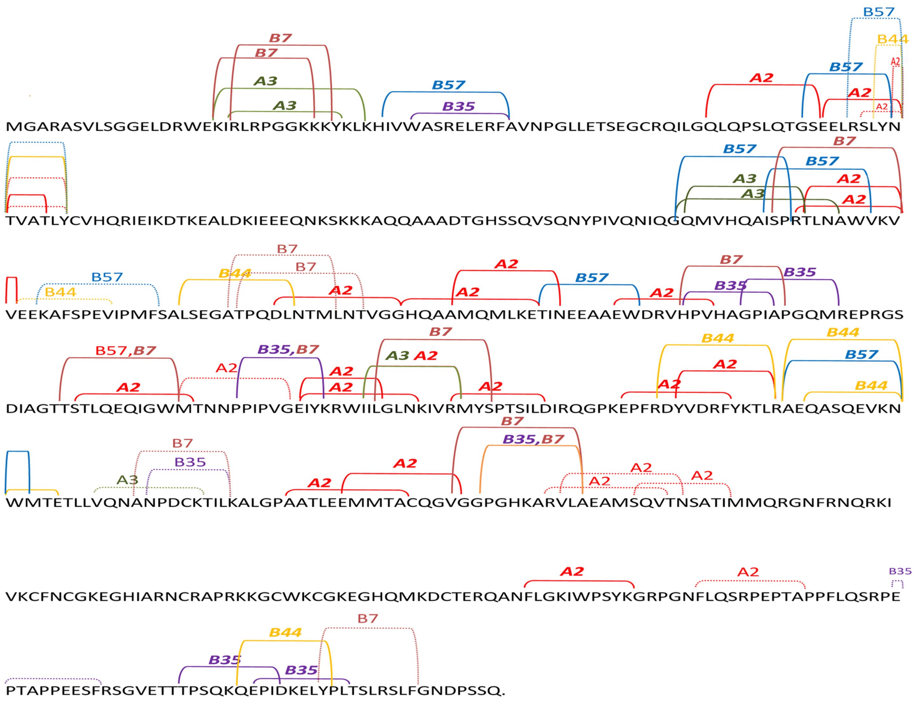

B.

atgggtgcgagagcgtcagtattaagcgggggagaattagatcgatgggaaaaaattcggttaaggccagggggaaagaa
aaaatataaattaaaacatatagtatgggcaagcagggagctagaacgattcgcagttaatcctggcctgttagaaacat
cagaaggctgtagacaaatactgggacagctacaaccatcccttcagacaggatcagaagaacttagatcattatataat
acagtagcaaccctctattgtgtgcatcaaaggatagagataaaagacaccaaggaagctttagacaagatagaggaaga
gcaaaacaaaagtaagaaaaaagcacagcaagcagcagctgacacaggacacagcagccaggtcagccaaaattacccta
tagtgcagaacatccaggggcaaatggtacatcaggccatatcacctagaactttaaatgcatgggtaaaagtagtagaa
gaggaaggctttcagcccagaagtgatacccatgttttcagcattatcagaaggagccaccccacaagatttaaacaccat
gctaaacacagtggggggacatcaagcagccatgcaaatgttaaaagagaccatcaatgaggaagctgcagaatgggata
gagtgcatccagtgcatgcagggcctattgcaccaggccagatgagagaaccaaggggaagtgacatagcaggaactact
agtacccttcaggaacaaataggatggatgacaaataatccacctatcccagtaggagaaatttataaaagatggataat
cctgggattaaataaaatagtaagaatgtatagccctaccagcattctggacataagacaaggaccaaaagaacccttta
gagactatgtagaccggttctataaaactctaagagccgagcaagcttcacaggaggtaaaaaattggatgacagaaacc
ttgttggtccaaaatgcgaacccagattgtaagactattttaaaagcattgggaccagcagctacactagaagaaatgat
gacagcatgtcagggagtgggaggacccggccataaggcaagagttttggctgaagcaatgagccaagtaacaaattcag
ctaccataatgatgcaaagaggcaattttaggaaccaaagaaagattgttaagtgtttcaattgtggcaaagaagggcac
atagccagaaattgcagggcccctaggaaaaagggctgttggaaatgtggaaaggaaggacaccaaatgaaagattgtac
tgagagacaggctaattttttagggaagatctggccttcctacaagggaaggccagggaattttcttcagagcagaccag
agccaacagccccaccatttcttcagagcagaccagagccaacagccccaccagaagagagcttcaggtctggggtagag
acaacaactccctctcagaagcaggagccgatagacaaggaactgtatcctttaacttccctcagatcactctttggcaa
cgacccctcgtcacaataa

C.

Wild type

peptide seq: AISPRTLNAW

(+)strand DNA seq: *gccatatcacctagaactttaaatgcatgg*

Mutant

peptide seq: AISPKTLNAW

(+)strand DNA seq: *gccatatcacctaaa*

Wild type

peptide seq: VPLDEDFRKY

(+)strand DNA seq: *gttcccttagatgaagacttcaggaagtat*

Mutant

peptide seq: VPLDKDFRKY

(+)strand DNA seq: *gttcccttagataaagacttcaggaagtat*

or

Wild type

peptide seq: VPLDEDFRKY

(+)strand DNA seq: *gttcccttagatgaagacttcaggaagtat*

Mutant

peptide seq: VPLDEDFKKY

(+)strand DNA seq: *gttcccttagatgaagacttcaagaagtat*

Wild type

peptide seq: FLGKIWPSHK

(+)strand DNA seq: *tttttagggaagatctggccttcctacaag*

peptide seq: FLGKIWPSHK

(+)strand DNA seq: *tttttaggaaagatctggccttcctacaag*

peptide seq: FLGKIWPSHK

(+)strand DNA seq: *tttttaggaaagatctggccttcctacaag*

Mutant

peptide seq: FLRKIWPSHK

(+)strand DNA seq: *tttttaagaaagatctggccttcctacaag*

Supplementary Figure 1
