# Supplementary material for: Positioning of APOBEC3G/F Mutational Hotspots in the Human Immunodeficiency Virus Genome Favors Reduced Recognition by CD8+ T Cells
Source: PLoS One. 2014 Apr 10;9(4):e93428. doi: 10.1371/journal.pone.0093428 (PMC3982959; doi:10.1371/journal.pone.0093428)
Supplement: Figure S2 — Map of all HIV-1 Bru isolate CTL epitopes across the viral proteome. HIV proteins are shown individually (A: Pol, B: Rev, C: Vif, D: Nef, E: Vpr, F: Env, G: Gag, H: Tat). The brackets indicate epitopes on the peptide sequence. Epitopes restricted to different HLA alleles are shown in different colors and the restricting HLA is indicated above the bracket. Broken lines display epitopes with no A3G/F hotspots and continuous lines show epitopes that harbor A3G/F hotspots. (PDF) [file pone.0093428.s002.pdf]

**A.**

tttttagggaagatctggccttcctacaagggaaggccagggaattttcttcagagcagaccagagccaacagcccca
catttcttcagagcagaccagagccaacagccccaccagaagagagcttcaggtctggggtagagacaacaactccctct
cagaagcaggagccgatagacaaggaactgtatcctttaacttccctcagatcactctttggcaacgacccctcgtcaca
ataaagataggggggcaactaaaggaagctctattagatacaggagcagatgatacagtattagaagaaatgagtttgcc
aggaagatggaaaccaaaaatgatagggggaattggaggttttatcaaagtaagacagtatgatcagatactcatagaaa
tctgtggacataaagctataggtacagtattagtaggacctacacctgtcaacataattggaagaaatctgttgactcag
attggttgcactttaaattttcccattagtcctattgaaactgtaccagtaaaattaaagccaggaatggatggcccaaa
agttaaacaatggccattgacagaagaaaaaataaaagcattagtagaaatttgtacagaaatggaaaaggaaggaaaa
tttcaaaaattgggcctgaaaatccatacaatactccagtatttgccataaagaaaaaagacagtactaaatggagaaaa
ttagtagatttcagagaacttaataagagaactcaagacttctgggaagttcaattaggaataccacatcccgcagggtt
aaaaaagaaaaaatcagtaacagtactggatgtgggtgatgcatattttcagttcccttagatgaagacttcaggaagt
atactgcatttaccatacctagtataaacaatgagacaccagggattagatatcagtacaatgtgcttccacagggatgg
aaaggatcaccagcaatattccaaagtagcatgacaaaaatcttagagccttttagaaaacaaaatccagacatagttat
ctatcaatacatggatgatttgtatgtaggatctgacttagaaatagggcagcatagaacaaaaatagaggagctgagac
aacatctgttgaggtggggacttaccacaccagacaaaaaacatcagaaagaacctccattcctttggatgggttatgaa
ctccatcctgataaatggacagtacagcctatagtgctgccagaaaaagacagctggactgtcaatgacatacagaagtt
agtgggaaaattgaattgggcaagtcagatttacccagggattaaagtaaggcaattatgtaaactccttagaggaacca
aagcactaacagaagtaataccactaacagaagaagcagagctagaactggcagaaaacagagagattctaaaagaacca
gtacatggagtgtattatgacccatcaaaagacttaatagcagaaatacagaagcaggggcaaggccaatggacatatca
aatttatcaagagccatttaaaaatctgaaaacaggaaaatatgcaagaacgaggggtgcccacactaatgatgtaaaac
aattaacagaggcagtgcaaaaaataaccacagaaagcatagtaatatggggaaagactcctaaatttaaactacccata
caaaaggaaacatgggaaacatggtggacagagtattggcaagccacctggattcctgagtgggagtttgtcaatacccc
tcctttagtgaaattatggtaccagttagagaaagaacccatagtaggagcagaaacgttctatgtagatggggcagcta
gcagggagactaaattaggaaaagcaggatatgttactaatagaggaagacaaaagttgtcaccctaactgacacaaca
aatcagaagactgagttacaagcaattcatctagctttgcaggattcgggattagaagtaaatatagtaacagactcaca
atatgcattaggaatcattcaagcacaaccagataaaagtgaatcagagttagtcaatcaaataatagagcagttaataa
aaaaggaaaaggtctatctggcatgggtaccagcacacaaaggaattggaggaaatgaacaagtagataaattagtcagt
gctggaatcaggaaagtactatttttagatggaatagataaggcccaagatgaacatgagaaatatcacagtaattggag
agcaatggctagtgattttaacctgccacctgtagtagcaaaagaaatagtagccagctgtgataaatgtcagctaaaag
gagaagccatgcatggacaagtagactgtagtccaggaatatggcaactagattgtacacatttagaaggaaaagttatc
ctggtagcagttcatgtagccagtggatatatagaagcagaagttattccagcagaaacagggcaggaaacagcatactt
tcttttaaaattagcaggaagatggccagtaaaaacaatacatacagacaatggcagcaatttcaccagtactacggtta
aggccgcctgttggtgggcgggaatcaagcaggaatttggaattccctacaatccccaaagtcaaggagtagtagaatct
atgaataaagaattaaagaaaattataggccaggtaagagatcaggctgaacatcttaagacagcagtacaaatggcagt
attcatccacaattttaaaagaaaaggggggattggggggtacagtgcaggggaaagaatagtagacataatagcaacag
acatacaaactaaagaattacaaaaacaaattacaaaaattcaaaattttcgggtttattacagggacagcagagatcca
ctttggaaaggaccagcaaagctcctctggaaaggtgaaggggcagtagtaatacaagataatagtgacataaaagtagt
gccaagaagaaaagcaaagatcattagggattatggaaaacagatggcaggtgatgattgtgtggcaagtagacaggatg
aggattag

**B.**

atggcaggaagaagcggagacagcgacgaagacctcctcaaggcagtcagactcatcaagtttctctatcaaagcaaccc
acctcccaaccccgaggggacccgacaggcccgaaggaatagaagaagaaggtggagagagagacagagacagatccatt
cgattagtgaacggatccttagcacttatctgggacgatctgcggagcctgtgcctcttcagctaccaccgcttgagaga
cttactcttgattgtaacgaggattgtggaacttctgggacgcagggggtgggaagccctcaaatattggtggaatctcc
tacagtattggagtcaggaactaaagaatag

**C.**

atggaaaacagatggcaggtgatgattgtgtggcaagtagacaggatgaggattagaacatggaaaagtttagtaaaaca
ccatatgtatgtttcagggaaagctaggggatggttttatagacatcactatgaaagccctcatccaagaataagttcag
aagtacacatcccactaggggatgctagattggtaataacaacatattggggtctgcatacaggagaaagagactggcat
ctgggtcagggagtctccatagaatggaggaaaaagagatatagcacacaagtagaccctgaactagcagaccaactaat
tcatctgtattactttgactgtttttcagactctgctataagaaaggccttattaggacatatagttagccctaggtgtg
aatatcaagcaggacataacaaggtaggatctctacaatacttggcactagcagcattaataacaccaaaaaagataaag
ccacctttgcctagtgttacgaaactgacagaggatagatggaacaagccccagaagaccaagggccacagagggagcca
cacaatgaatggacactag

**D.**

atgggtggcaagtggtcaaaaagtagtgtggttggatggcctactgtaagggaaagaatgagacgagctgagccagcagc
agatgggtgggagcagcatctcgagacctggaaaaacatggagcaatcacaagtagcaatacagcagctaccaatgctg
cttgtgcctggctagaagcacaagaggaggaggaggtgggttttccagtcacacctcaggtacctttaagaccaatgact
tacaaggcagctgtagatcttagccactttttaaaagaaaaggggggactggaagggctaattcactcccaacgaagaca
agatatccttgatctgtggatctaccacacacaaggctacttccctgattggcagaactacacaccagggccaggggtca
gatatccactgacctttggatggtgctacaagctagtaccagttgagccagataaggtagaaggaggccaataaaggagag
aacaccagcttgttacaccctgtgagcctgcatggaatggatgaccctgagagagaagtgttagagtggaggtttgacag
ccgcctagcatttcatcacgtggcccgagagctgcatccggagtacttcaagaactgctga

**E.**

atggaacaagccccagaagaccaagggccacagagggagccacacaatgaatggacactagagcttttagaggagcttaa
gaatgaagctgttagacattttcctaggatttggctccatggcttagggcaacatatctatgaaacttatggggatactt
gggcaggagtggaagccataataagaattctgcaacaactgctgtttatccatttcagaattgggtgtcgacatagcaga
ataggcgttactcaacagaggagagcaagaaatggagccagtagatcctag

**F.**

atgagagtgaaggagaaatatcagcacttgtggagatgggggtggaaatggggcaccatgctccttgggatattgatgat
ctgtagtgctacagaaaaattgtgggtcacagtctattatggggtacctgtgtggaaggaagcaaccaccactctatttt
gtgcatcagatgctaaagcatatgatacagaggtacataatgtttgggccacacatgcctgtgtacccacagaccccaac
ccacaagaagtagtattggtaaatgtgacagaaaattttaacatgtggaaaaatgacatggtagaacagatgcatgagga
tataatcagtttatgggatcaaagcctaaagccatgtgtaaaattaaccccactctgtgttagtttaaagtgcactgatt
tggggaatgctactaataccaatagtagtaataccaatagtagtagcggggaaatgatgatggagaaaggagagataaaa
aactgctctttcaatatcagcacaagcataagaggtaaggtgcagaaagaatatgcatttttttataaacttgatataat
accaatagataatgatactaccagctatacgttgacaagttgtaacacctcagtcattacacaggcctgtccaaaggtat
cctttgagccaattcccatacattattgtgccccggctggttttgcgattctaaaatgtaataataagacgttcaatgga
acaggaccatgtacaaatgtcagcacagtacaatgtacacatggaattaggccagtagtatcaactcaactgctgttgaa
tggcagtctagcagaagaagaggtagtaattagatctgccaatttcacagacaatgctaaaaccataatagtacagctga
accaatctgtagaaattaattgtacaagacccaacaacaatacaagaaaaagtatccgtatccagaggggaccagggaga
gcatttgttacaataggaaaaataggaaatatgagacaagcacattgtaacattagtagagcaaaatggaatgccacttt
aaaacagatagctagcaaattaagagaacaatttggaaataataaaacaataatctttaagcaatcctcaggaggggacc
cagaaattgtaacgcacagttttaattgtggaggggaatttttctactgtaattcaacacaactgtttaatagtacttgg
tttaatagtacttggagtactgaagggtcaaataacactgaaggaagtgacacaatcacactcccatgcagaataaaaca
atttataaacatgtggcaggaagtaggaaaagcaatgtatgcccctcccatcagcggacaaattagatgttcatcaaata
ttacagggctgctattaacaagagatggtggtaataacaacaatgggtccgagatcttcagacctggaggaggagatatg
agggacaattggagaagtgaattatataaatataaagtagtaaaaattgaaccattaggagtagcacccaccaaggcaaa
gagaagagtggtgcagagagaaaaaagagcagtgggaataggagctttgttccttgggttcttgggagcagcaggaagca
ctatgggcgcacggtcaatgacgctgacggtacaggccagacaattattgtctggtatagtgcagcagcagaacaatttg
ctgagggctattgaggcgcaacagcatctgttgcaactcacagtctggggcatcaagcagctccaggcaagaatcctggc
tgtggaaagatacctaaaggatcaacagctcctggggatttggggttgctctggaaaactcatttgcaccactgctgtgc
cttggaatgctagttggagtaataaatctctggaacagatttggaataacatgacctggatggagtgggacagagaaatt
aacaattacacaagcttaatacattccttaattgaagaatcgcaaaaccagcaagaaaagaatgaacaagaattattgga
attagataaatgggcaagtttgtggaattggtttaacataacaaattggctgtggtatataaaaatattcataatgatag
taggaggcttggtaggtttaagaatagtttttgctgtactttctatagtgaatagagttaggcagggatattcaccatta
tcgtttcagacccacctcccaaccccgaggggacccgacaggcccgaaggaatagaagaagaaggtggagagagagacag
agacagatccattcgattagtgaacggatccttagcacttatctgggacgatctgcggagcctgtgcctcttcagctacc
accgcttgagagacttactcttgattgtaacgaggattgtggaacttctgggacgcagggggtgggaagccctcaaatat
tggtggaatctcctacagtattggagtcaggaactaaagaatagtgctgttagcttgctcaatgccacagccatagcagt
agctgaggggacagatagggttatagaagtagtacaaggagcttgtagagctattcgccacatacctagaagaataagac
agggcttggaaaggattttgctataa

**G.**

atgggtgcgagagcgtcagtattaagcgggggagaattagatcgatgggaaaaaattcggttaaggccagggggaaagaa
aaaatataaattaaaacatatagtatgggcaagcagggagctagaacgattcgcagttaatcctggcctgttagaaacat
cagaaggctgtagacaaatactgggacagctacaaccatcccttcagacaggatcagaagaacttagatcattatataat
acagtagcaaccctctattgtgtgcatcaaaggatagagataaaagacaccaaggaagctttagacaagatagaggaaga
gcaaaacaaaagtaagaaaaaagcacagcaagcagcagctgacacaggacacagcagccaggtcagccaaaattacccta
tagtgcagaacatccaggggcaaatggtacatcaggccatatcacctagaactttaaatgcatgggtaaaagtagtagaa
gagaaggctttcagcccagaagtgatacccatgttttcagcattatcagaaggagccaccccacaagatttaaacaccat
gctaaacacagtggggggacatcaagcagccatgcaaatgttaaaagagaccatcaatgaggaagctgcagaatgggata
gagtgcatccagtgcatgcagggcctattgcaccaggccagatgagagaaccaaggggaagtgacatagcaggaactact
agtacccttcaggaacaaataggatggatgacaaataatccacctatcccagtaggagaaatttataaaagatggataat
cctgggattaaataaaatagtaagaatgtatagccctaccagcattctggacataagacaaggaccaaaggaaccctttaa
gagactatgtagaccggttctataaaactctaagagccgagcaagcttcacaggaggtaaaaaattggatgacagaaacc
ttgttggtccaaaatgcgaacccagattgtaagactattttaaaagcattgggaccagcagctacactagaagaaatgat
gacagcatgtcagggagtgggaggacccggccataaggcaagagttttggctgaagcaatgagccaagtaacaaattcag
ctaccataatgatgcaaagaggcaattttaggaaccaaagaaagattgttaagtgtttcaattgtggcaaagaagggcac
atagccagaaattgcagggcccctaggaaaaagggctgttggaaatgtggaaaggaaggacaccaaatgaaagattgtac
tgagagacaggctaattttttagggaagatctggccttcctacaagggaaggccagggaattttcttcagagcagaccag
agccaacagccccaccatttcttcagagcagaccagagccaacagccccaccagaagagagcttcaggtctggggtagag
acaacaactccctctcagaagcaggagccgatagacaaggaactgtatcctttaacttccctcagatcactctttggcaa
cgacccctcgtcacaataa

**H.**

atggagccagtagatcctagactagagccctggaagcatccaggaagtcagcctaaaactgcttgtaccacttgctattg
taaaaagtgttgctttcattgccaagtttgtttcacaacaaaagccttaggcatctcctatggcaggaagaagcggagac
agcgacgaagacctcctcaaggcagtcagactcatcaagtttctctatcaaagcaacccacctcccaaccccgaggggac
ccgacaggcccgaaggaatag

**Supplementary Figure 2**
