# Supplementary figures and images for: Positioning of APOBEC3G/F Mutational Hotspots in the Human Immunodeficiency Virus Genome Favors Reduced Recognition by CD8+ T Cells
Source: PLoS One. 2014 Apr 10;9(4):e93428. doi: 10.1371/journal.pone.0093428 (PMC3982959; doi:10.1371/journal.pone.0093428)

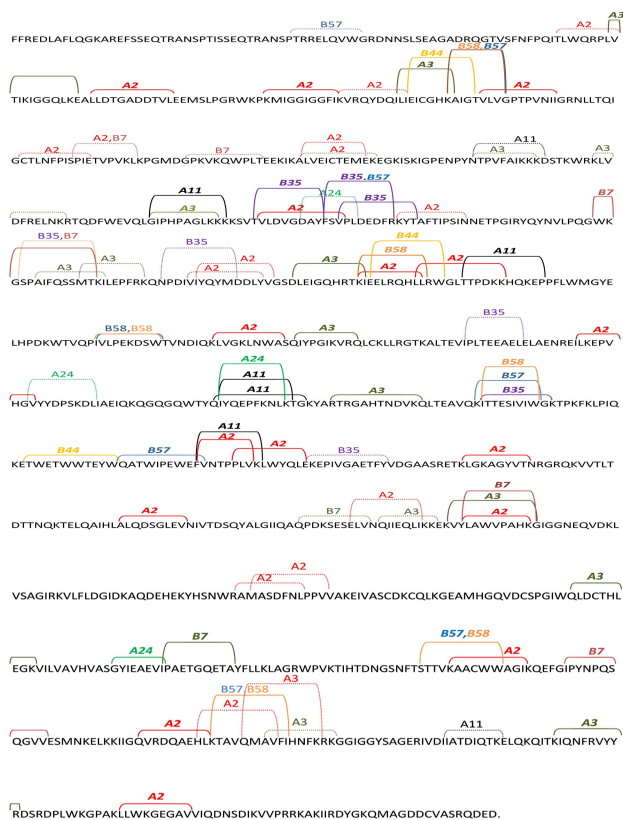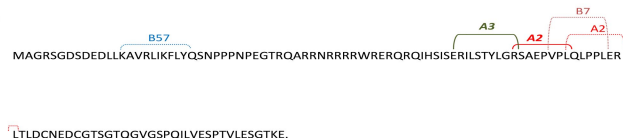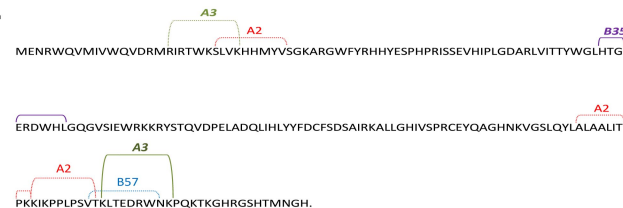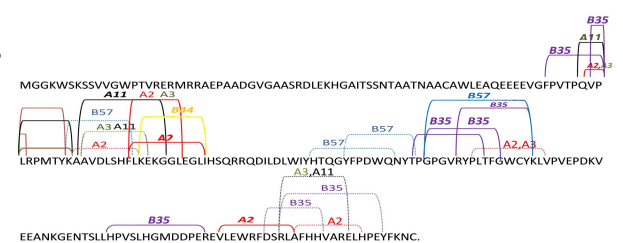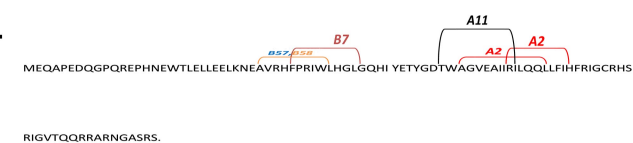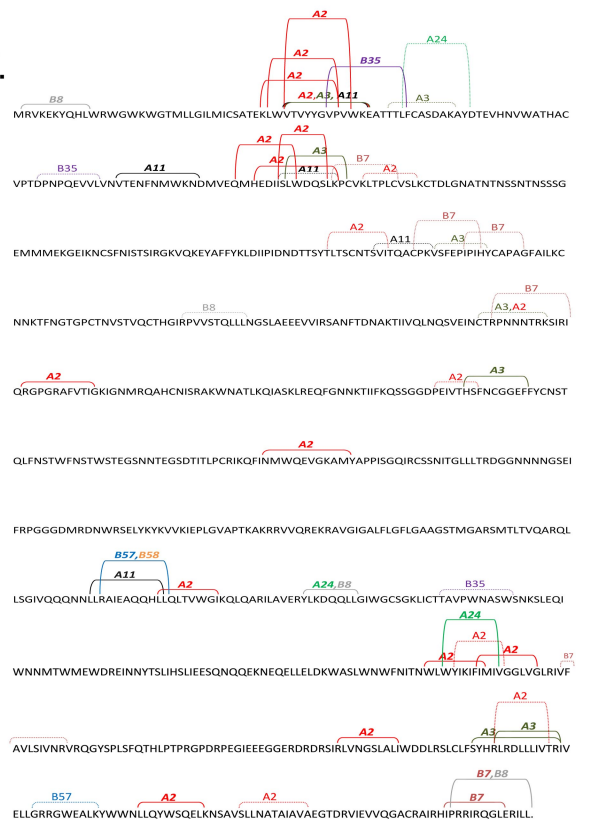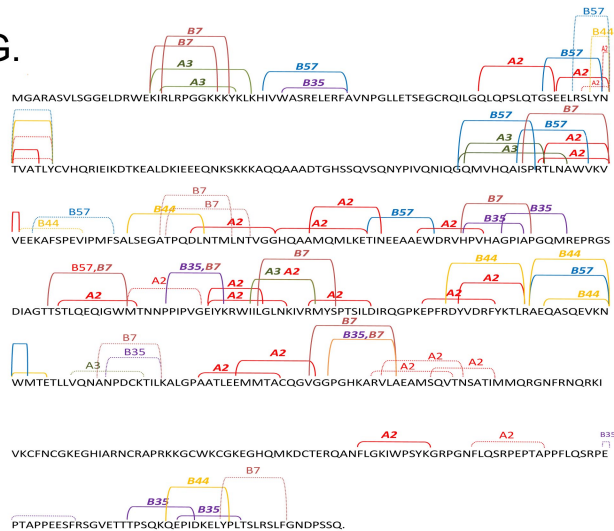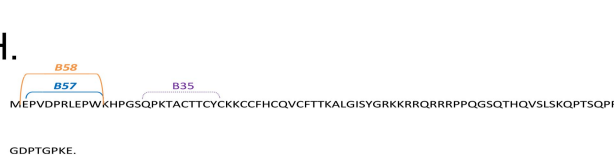

Supplement: Figure S3 — Map of the viral genomic sequences that encode CTL epitopes in the HIV-1 Bru isolate plus-sense coding DNA. Sequence of each gene is shown (A: Pol, B: Rev, C: Vif, D: Nef, E: Vpr, F: Env, G: Gag, H: Tat). Colors differentiate sequences encoding CTL epitopes from the surrounding DNA. Colors do not otherwise correspond to any common features amongst genes or epitopes. (PDF) [file pone.0093428.s003.pdf]
