# Supplementary material for: Positioning of APOBEC3G/F Mutational Hotspots in the Human Immunodeficiency Virus Genome Favors Reduced Recognition by CD8+ T Cells
Source: PLoS One. 2014 Apr 10;9(4):e93428. doi: 10.1371/journal.pone.0093428 (PMC3982959; doi:10.1371/journal.pone.0093428)
Supplement: Table S1 — Relative enrichment of A3G/F hotspot inside/outside CTL epitopes in HIV genes. For each HIV gene, the frequency of A3G/F hotspots (either all hotspots: GGG, GGA, GGT, GAA, or GGG alone) was calculated in genomic sequences that encode for CTL epitopes and those that do not encode for CTL epitopes. Normalized frequencies were calculated by taking into account the total nucleotide length of the sequence. For each HIV gene, the normalized frequency of A3G/F hotspots inside sequences that encode CTL motifs was divided by the normalized frequency of A3G/F hotspots in sequences that do not encode CTL epitopes to generate a Ratio (R-value). The average of R-values was determined and each R-value was divided by the average to obtain the index value and allow for determination of the relative enrichment of hotspots within the CTL epitopes of each gene, as compared to the average ratio of hotspots inside to outside CTL epitopes. An index value >1 indicates relative enrichment. (PDF) [file pone.0093428.s004.pdf]

Supplementary Table 1.

Genes	Normalized number of hotspots inside CTL-epitopes	Normalized number of GGG hotspots inside CTL-epitopes	Normalized number of hotspots outside CTL-epitopes	Normalized number of GGG hotspots outside CTL-epitopes	Ratio of all hotspots Inside:outside epitopes	Index value	Ratio of GGG hotspots Inside:outside epitopes	Index value
Vpr	0.1041	0.0277	0.0884	0.0204	1.17	1.394	1.36	1.119
Pol	0.0924	0.023	0.0794	0.00883	1.16	1.382	2.60	2.139
Gag	0.0864	0.0233	0.0813	0.0143	1.06	1.263	1.63	1.341
Vif	0.0774	0.0202	0.0992	0.0177	0.78	0.929	1.14	0.938
Nef	0.0839	0.0272	0.0944	0.0111	0.889	1.059	2.45	2.016
Env	0.070	0.0176	0.0991	0.0226	0.706	0.841	0.779	0.641
Rev	0.094	0.00556	0.1345	0.0409	0.699	0.833	0.136	0.111
Tat	0.0363	0	0.1041	0.0208	0.349	0.416	<0.291	0.239
Vpu	0.0701	0	0.952	0.0317	0.736	0.877	<0.553	0.455
